# Microstructural grey matter alterations in patients with behavioural variant frontotemporal dementia

**DOI:** 10.1093/braincomms/fcaf427

**Published:** 2025-11-03

**Authors:** Behnaz Akbarian, Kilian Hett, Tony Phan, Jayden J Lee, James Eaton, Manus J Donahue, R Ryan Darby

**Affiliations:** Department of Biomedical Engineering, Vanderbilt University, Nashville, TN 37235, USA; Department of Neurology, Vanderbilt University Medical Center, Nashville, TN 37232, USA; Department of Neurology, Vanderbilt University Medical Center, Nashville, TN 37232, USA; Department of Neurology, Vanderbilt University Medical Center, Nashville, TN 37232, USA; Department of Neurology, Vanderbilt University Medical Center, Nashville, TN 37232, USA; Department of Neurology, Vanderbilt University Medical Center, Nashville, TN 37232, USA; Department of Biomedical Engineering, Vanderbilt University, Nashville, TN 37235, USA; Department of Neurology, Vanderbilt University Medical Center, Nashville, TN 37232, USA

**Keywords:** frontotemporal dementia, brain volume, texture analysis, disease progression, early diagnosis

## Abstract

Behavioural variant frontotemporal dementia is characterized by progressive changes to personality and behaviour, yet early detection and disease staging remain challenging. Current clinical neuroimaging relies on visual assessment of atrophy patterns, which may overlook subtle structural changes. While volumetric analysis has improved the ability to detect neurodegeneration and track disease progression, it may lack the sensitivity to identify microstructural alterations that precede frank atrophy. Texture analysis, a quantitative approach that evaluates the spatial regularity of grey matter density, has demonstrated promise in detecting early microstructural changes in Alzheimer’s disease and distinguishing frontotemporal dementia subtypes. However, its potential as a biomarker for early behavioural variant frontotemporal dementia detection and disease staging remain unexplored. This study evaluates the potential of autocorrelation-based texture features as biomarkers for detecting early-stage behavioural variant frontotemporal dementia and tracking disease progression, comparing their sensitivity and specificity to regional brain volume. We analysed structural MRI scans from behavioural variant frontotemporal dementia patients with mild (*n* = 21, 61.5 ± 8.5 years, 4.7% female) and moderate dementia (*n* = 11, 64.4 ± 9.3, 18.1% female) alongside healthy controls (*n* = 33, 63.1 ± 7.9 years; 36.3% female). Texture and volumetric measures were extracted from frontotemporal regions implicated in behavioural variant frontotemporal dementia pathology. First, we compared these features between healthy controls and patients with mild dementia to identify regions relevant for early diagnosis. Second, we compared patients with mild versus moderate dementia to identify regions linked to disease stage. Analyses were performed both within a composite frontotemporal region of interest and within 160 frontotemporal subregions. We applied false discovery rate correction for multiple comparisons. Microstructural abnormalities captured by texture analysis and volumetric reductions were significantly lower in patients with mild dementia compared to healthy controls within the composite frontotemporal and many subregions (*P_FDR_*  *<* 0.05). In moderate dementia, texture features detected alterations in composite frontotemporal (*P_FDR_*  *<* 0.05) and subregions, including the anterior cingulate, insula and orbitofrontal cortices (*P_FDR_*  *<* 0.05) even when volumetric differences were absent. This study demonstrates that texture-based MRI metrics provide a sensitive measure of microstructural alterations in behavioural variant frontotemporal dementia, detecting some disease-related changes even in regions without detectable volumetric reductions. While volumetric measures are effective for identifying individuals with early-stage disease, texture analysis may offer increased sensitivity for tracking disease progression. Longitudinal studies are needed to validate the predictive value of texture features for clinical decline in behavioural variant frontotemporal dementia.

## Teaser

Akbarian *et al*. report that texture analysis of MRI detects subtle microstructural grey matter alterations in patients with behavioural variant frontotemporal dementia. These alternations are observed even when volumetric differences are absent, indicating that texture features may serve as a complementary biomarker for early diagnosis and for tracking disease progression.

## Graphical Abstract

**Graphical Abstract:**
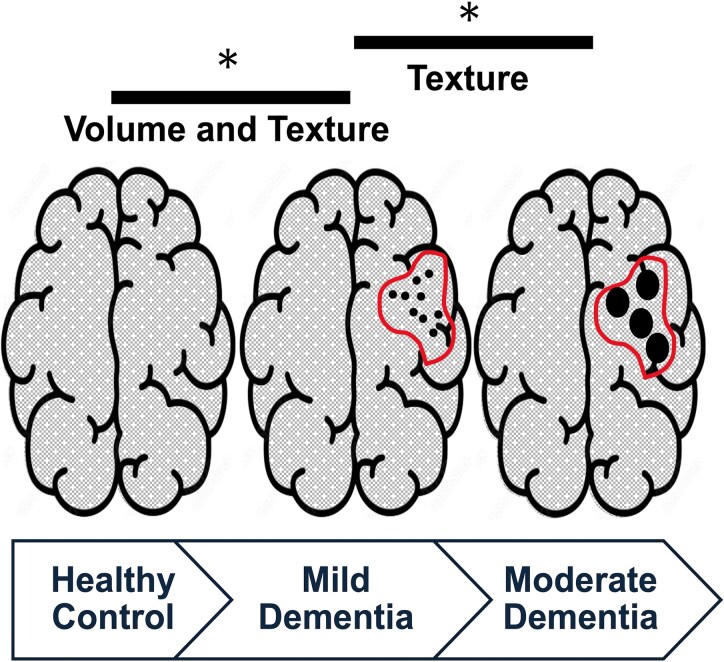

## Introduction

Frontotemporal dementia (FTD) is a heterogeneous group of neurodegenerative disorders that primarily affect the frontal and temporal lobes of the brain, leading to progressive changes in behaviour, personality, movement and language.^1^ Behavioural variant FTD (bvFTD) is the most common phenotype, characterized by prominent changes in social behaviour, personality, emotions, motivation and executive functions. Early diagnosis of bvFTD and prediction of its progression remain significant clinical challenges.^2^ Early detection of neurodegenerative diseases is important to guide patients and families towards appropriate therapies.^3^ Additionally, appropriately staging dementia is important for guiding clinical management decisions,^4^ including personalized care and the selection of pharmacologic treatments suited to different stages of disease severity.^5-7^

Neuroimaging, specifically structural MRI, is a particularly valuable biomarker of neurodegeneration and disease progression that improves diagnostic confidence in bvFTD.^8^ However, current clinical assessments predominantly rely on visual inspection to detect neuroimaging abnormalities, which depends on the experience of the reader and may miss subtle changes, leading to missed diagnoses. Quantitative approaches that measure abnormal brain volumes offer several advantages over visual inspection in FTD, including early detection and diagnosis,^9,10^ as well as tracking clinical changes and disease progression over time.^11-23^ However, brain volume measurements may lack the sensitivity to detect subtle structural changes that could precede frank volume loss.

Texture analysis is an approach that characterizes the microstructural pattern and regularity of grey matter density rather than measuring its overall volume. In the context of neurodegenerative diseases such as FTD, we hypothesize that neurodegeneration leads to subtle microstructural changes that may be more sensitive indicators than volume loss. Supporting this hypothesis, prior studies in patients with Alzheimer’s disease,^24,25^ found that texture analysis detected microstructural changes in grey matter earlier than volume-based measures,^24,26^ predicted the progression from mild cognitive impairment (MCI) to Alzheimer’s disease,^27,28^ and correlated with tau PET tracer binding,^29^ linking changes in texture to neuropathology. Additionally, texture analysis can distinguish between different FTD syndromes^30^ and has been shown to be superior to volume-based measures in differentiating semantic-variant primary progressive aphasia (svPPA) from Alzheimer’s disease.^31^ However, no prior studies have investigated whether texture analysis can detect microstructural changes in bvFTD patients in the early stages of the disease and throughout disease progression, nor has it been compared to measurable volume loss.

In this study, we leverage standard volumetric assessments alongside advanced texture analysis to test the hypothesis that texture features provide greater sensitivity than volume in detecting early-stage bvFTD and distinguishing between clinical stages of disease severity. Finally, we compare the performance of both approaches using receiver operating characteristic (ROC) curve analysis.

## Materials and methods

### Participants

This study included 32 patients clinically diagnosed with bvFTD (mean age: 62.5 ± 8.7 years) and 33 age-matched healthy controls (HCs) (mean age: 63.1 ± 7.9 years). Patients were recruited from the Behavioural and Cognitive Neurology clinics in the Department of Neurology, Vanderbilt University Medical Center between July 2018 and March 2023. Patients were referred to the clinic for a variety of concerns, including cognitive, behavioural or neuropsychiatric changes. HCs were recruited from the Vanderbilt Memory and Alzheimer’s Center at Vanderbilt University Medical Center as part of the Tennessee Alzheimer’s Project between November 2021 and October 2022. Patient consent was obtained according to the Declaration of Helsinki and approved by the Vanderbilt University Institutional Review Board. At enrolment, patients underwent a general medical and neurological evaluation. The diagnosis of bvFTD was confirmed in each patient by a behavioural neurologist (R.R.D.) in accordance with international research criteria.^8^ Inclusion criteria required that patients have a reliable study informant present during the study visit, defined as an individual who spends a significant period of time (i.e. in-person interactions greater than or equal to two times per week) with the patient, and an MRI scan that passed quality control. The dementia stage was rated by the study neurologist as mild dementia or moderate dementia based on clinical assessment and evaluation of functional independence in activities of daily living. In addition, the Montreal Cognitive Assessment (MoCA) was administered to all patients as a general measure of cognitive function.^32^ Patients’ clinical information is summarized in Table 1.

**Table 1:** Demographic and clinical information of patients with bvFTD and HC

All patients with bvFTD versus HC			
Demographic information	HC (*n* = 33)	bvFTD (*n* = 32)	*P*-value*
Age (years: mean ± SD)	63.1 ± 7.9	62.5 ± 8.7	0.75^a^
Sex (% female)	36.3	9.3	0.0012^b^
Race/ethnicity (# (%))			0.85^b^
BorAA. Non-Hispanic	3 (9.0)	2 (6.25)	-
White. Non-Hispanic	30 (90.9)	29 (90.62)	-
White/unknown	0 (0)	1 (3.1)	-
Patients with mild dementia versus patients with moderate dementia			
Clinical variable	Mild dementia (*n* = 21)	Moderate dementia (*n* = 11)	*P*-value**
Age (years: mean ± SD)	61.5 ± 8.5	64.4 ± 9.3	0.31^a^
Sex (% female)	4.7	18.1	0.96^b^
MoCA score	21.0 ± 5.4	16.2 ± 6.4	0.06^a^
Race/ethnicity (# (%)).			0.69^b^
BorAA. Non-Hispanic	2 (9.5)	0 (0)	-
White. Non-Hispanic	19 (90.4)	11 (100)	-
White/unknown	1 (4.7)	0 (0)	-
Education [# (%)].			0.44^b^
High school degree or equivalent	5 (23.8)	1 (9.0)	-
Some college, no degree	5 (23.8)	4 (36.3)	-
College degree	7 (33.3)	2 (18.1)	-
Advanced degree	4 (19.0)	4 (36.6)	-
Informant relationship [# (%)]			0.68^b^
Spouse	17 (80.9)	10 (90.9)	-
Child	3 (14.2)	1 (9.0)	-
Other	1 (4.7)	0 (0)	-

BorAA, Black or African American.

^a^Wilcoxon rank-sum test.

^b^Chi-squared test.

^*^Statistics and *P*-values compare the demographic information across the patients with bvFTD and healthy control.

^**^Statistics and *P*-values compare the clinical variables across the patients with mild dementia and patients with moderate dementia.

### MRI data acquisition

T1-weighted images were acquired using a 3T MRI scanner (Philips Healthcare, Best, The Netherlands) with dual-channel body coil transmission and 32-channel phased-array head coil reception. A whole-brain 3D magnetization-prepared rapid acquisition gradient echo (MPRAGE) sequence acquired in the sagittal plane was employed with the following parameters: *TR* = 8.1 ms, *TE* = 3.7 ms, *flip angle* = 8°, *field-of-view* = 256 × 256 mm, slice thickness = 1 mm, spatial resolution = 1 × 1 × 1 mm^3^ and number of slices = 150. Sagittal 3D Accelerated MPRAGE (MSV21) protocol was used for the HC subjects with the following parameters: *TR* = 6.5 ms, *TE* = 2.9 ms, *flip angle* = 9°, *field-of-view* = 256 × 256 mm, slice thickness = 1 mm, spatial resolution 1 × 1 × 1 mm^3^ and number of slices = 211.

### Volumetric analysis

Volumetric analysis was performed using voxel-based morphometry (VBM) analysis. VBM was performed on the acquired T1-weighted images to investigate regional differences in brain structure across our study cohort.^33^ VBM analysis was conducted using the SPM12 software package (http://www.fil.ion.ucl.ac.uk/spm/software/spm12). The analysis pipeline included tissue segmentation into grey matter, white matter and CSF; normalization to the Montreal Neurological Institute (MNI) template space; modulation to preserve tissue volume;^34^ and spatial smoothing using an 8-mm full-width at half-maximum Gaussian kernel. Next, images were divided by intracranial volume (grey matter + white matter + CSF) to account for individual differences in overall brain size, enabling a more precise investigation of regional volumetric changes.^35^ Finally, grey matter intensities were extracted and summed to calculate the total partial volume within each region of interest (ROI).

### Texture analysis

#### Preprocessing

Preprocessing of T1-weighted MRI images was performed using advanced normalization tools (ANTs) (v2.3.4) and FreeSurfer (v7.2) to ensure data quality and consistency before texture analysis.^36^ The pipeline consisted of three main steps **(**Supplementary Fig. 1). First, bias field correction was applied using the N4 algorithm in ANTs to reduce intensity inhomogeneities. Second, skull stripping was performed using FreeSurfer’s *recon-all* command to remove non-brain tissues such as scalp, dura, bone and fat. All skull-stripped images were visually inspected to ensure accurate brain extraction. Following skull stripping, each subject’s T1-weighted image was nonlinearly registered to the MNI152 1 mm template using ANTs. The skull-stripped T1w image served as the moving image, and the MNI template served as the fixed image. A symmetric diffeomorphic (SyN) transformation was applied to warp the moving image to template space. The resulting transformations were then applied to the T1w image and the labelled atlas (HCPex) to generate both the subject image in template space and the label map in subject space, ensuring accurate alignment for subsequent ROI-based texture analysis.

#### Feature extraction

**Figure 1:**
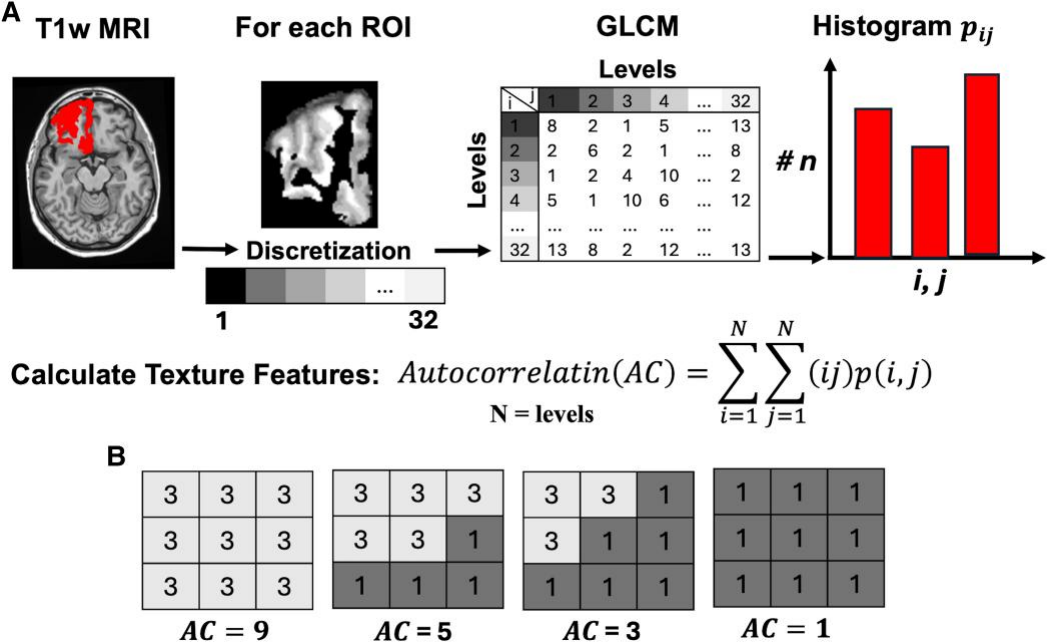
**Texture analysis.** (**A**) Texture analysis pipeline for detecting microstructural measure in grey matter. The pipeline includes (i) intensity normalization within each ROI using the dynamic limited method (μ ± 3σ), (ii) uniform quantization for intensity discretization with 32 levels, (iii) computation of the GLCM to capture spatial relationships between voxel intensities and (iv) extraction of texture features from GLCM histograms. (**B**) Illustration of the relationship between neuronal loss and autocorrelation-based texture features. Each 3 × 3 matrix represents a simplified model of a brain image after discretization with a level of 3, where voxel intensities are assigned values of 1 (lower volume, representing neuronal loss) or 3 (higher volume, representing preserved tissue). Higher autocorrelation values correspond to more homogeneous regions (more hyperintense areas in MRI), whereas lower autocorrelation values indicate disrupted tissue structure associated with neuronal degeneration (more hypointense regions in MRI). For simplicity, to illustrate the concept, autocorrelation values were calculated based on voxel intensity adjustments in the vertical and horizontal directions with a distance of 1.

We used texture analysis to evaluate microstructural changes of grey matter. Texture analysis quantified the variations in pixel intensities within an MRI image, such as T1-weighted images, to identify unique textural features that may not be discernible visually.^22^ To calculate texture features, first intensity values of voxels inside each ROI was normalized by using dynamic limited method (μ±3σ).^37^ Next, the uniform quantization for intensity discretization was applied within each ROI, reducing the number of distinct intensity values in an image to a specified value, called levels. We selected the 32 as the level for extracting texture features as guided by prior literature recommendations.^23^ The next step was to calculate the grey level-cooccurrence matrix (GLCM).^24^ The GLCM is a statistical method that quantifies how often different combinations of pixel intensity values occur. Finally, texture features were calculated based on histogram values in GLCM (Fig. 1A). In this study, we calculated the autocorrelation- based texture feature. While many different texture features can be extracted, we focused here on autocorrelation, which was sensitive to cerebral degeneration in amyotrophic lateral sclerosis,^38^ Alzheimer’s disease^39^ and svPPA^31^ in prior studies, even in the absence of detectable volumetric changes.^31,40^

#### Autocorrelation-based texture feature

Autocorrelation is a statistical texture feature that quantifies the spatial dependencies of voxel intensities within an image. It measures the similarity of intensity values across neighbouring voxels, taking into account both intensity values and their spatial arrangement.^41,42^ Higher autocorrelation values indicate a more homogeneous texture; however, regions with overall atrophy (i.e. globally lower intensity values) do not necessarily exhibit high autocorrelation, because the measure is scaled by voxel intensity and considers local variations. Consequently, brain regions with microstructural disruption or heterogeneous tissue composition typically show lower autocorrelation values. Figure 1B demonstrates this hypothetical relationship between neurodegeneration and autocorrelation values.

### ROI definition

Our approach involved two levels of analysis. First, we examined texture and volumetric changes within a composite frontotemporal ROI, created by combining 160 ROIs within frontal and temporal regions based on the Glasser *et al.* parcellation.^20^ Second, we conducted a more granular analysis by evaluating these 160 subregions individually to capture localized patterns of neuronal degeneration. Supplementary Fig. 2 illustrates the selected ROIs for analysis.

### Statistical analysis

All statistical analyses were conducted using MATLAB 2023a.

#### Group differences in demographic measures

We compared the demographic information between patients with bvFTD and age-matched HCs. Patients with bvFTD were further classified as having mild or moderate dementia severity. The Wilcoxon rank-sum test was used to compare continuous variables (age, MoCA), while Fisher’s exact test was applied to compare categorical variables (sex, race/ethnicity, education level and caregiver relationship).

#### Group differences in brain volume and texture in bvFTD with mild, moderate dementia and controls

To test whether volumetric or texture abnormalities differ between bvFTD patients at different disease stages, we categorized bvFTD patients into two groups: mild (*n* = *21*) and moderate dementia (*n* = *11*), along with cognitively normal controls (*n* = *33*). Analyses were restricted to brain regions within frontal and temporal lobes, first using a composite frontotemporal ROI and then across 160 subregions within the frontotemporal cortex.

For each ROI, we performed an analysis of covariance (ANCOVA) with brain volume as the response variable, group (mild dementia versus moderate dementia versus cognitively normal controls) as the independent variable of interest, and age and sex as covariates. Similarly, ANCOVA was applied to test for group-level differences in texture, with autocorrelation as the response variable and group (mild dementia versus moderate dementia versus cognitively normal controls) as the independent variable of interest, controlling for age and sex. To account for multiple comparisons across 160 ROIs, we applied false discovery rate (FDR) corrections. We also calculated and plotted effect sizes for volume and autocorrelation, as well as their difference (volume—autocorrelation) to assess which metric demonstrated stronger group-level separation between HC and mild bvFTD, and between mild and moderate bvFTD, in each region.

In all figures, for visualization purposes only, raw values in patients were standardized to z-scores relative to the mean and standard deviation of the HC group. All statistical analyses were conducted on the original, non-standardized data.

#### Comparison between regional brain volume and autocorrelation-based texture feature models

To assess whether autocorrelation-based texture features or regional brain volume can quantitatively differentiate between bvFTD patients with mild dementia and HCs, as well as between bvFTD patients with mild and moderate dementia, we conducted ROC curve analysis. This approach enabled us to determine whether the neuroimaging-derived predicted group membership aligned with the actual clinical classification. The analysis was conducted using the composite frontotemporal ROI. The area under the curve (AUC), sensitivity and specificity were calculated for both texture, volume and a combination of texture and volume measures. Leave-one-out cross-validation utilized to evaluate the model’s performance. The optimal threshold was calculated based on ROC curve. To compare the performance of volume and autocorrelation-based texture measures, we compared their AUCs using DeLong’s test.^43^

#### The relationship between texture and volume

Finally, we investigated the linear relationship between autocorrelation-based texture features and brain volume, accounting for potential confounding factors. We performed linear regressions with regional brain volumes as the response variable, autocorrelation-based texture features as the variable of interest and age, and sex, as covariates. Multiple comparisons were controlled using FDR. For this analysis, we included both HCs and bvFTD patients. To account for differences between groups, we repeated this analysis by including group (healthy versus bvFTD) as a covariate.

## Results

### Demographic and clinical characteristics of participants

Clinical and demographic information is summarized in Table 1. None of the clinical or demographic variables were statistically different between patients with mild dementia and those with moderate dementia; however, the difference in MoCA scores between these groups approached significance (*P* = 0.06).

### Group differences in brain volume and texture between bvFTD with mild dementia and healthy controls

#### Composite frontotemporal ROI

**Figure 2:**
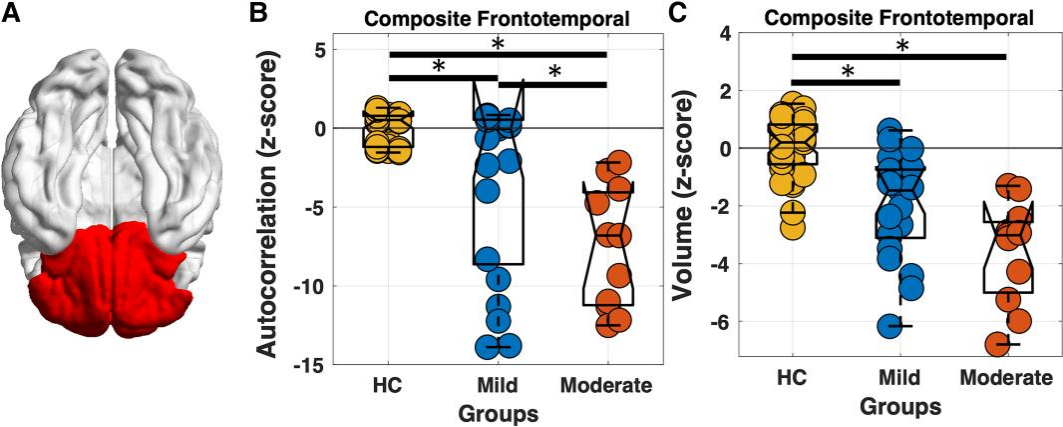
**Group comparison of neuroimaging measures.** (**A**) Brain regions highlighted in red represent the composite frontotemporal region of interest. (**B**) Comparison of autocorrelation-based texture feature between HCs (HC, *n* = 33), mild (*n* = 21) and moderate (*n* = 11) bvFTD patients in the frontal-temporal region. (**C**) Comparison of volume between HC, mild, and moderate bvFTD patients in the frontal-temporal region. Statistical comparisons were corrected for multiple testing (*P_FDR_* < 0.05, ANCOVA adjusted for age and sex). The figure visually represents lower autocorrelation-based texture feature and volume in patients with mild dementia compared to HC, and lower autocorrelation-based texture feature in patients with moderate dementia compared to mild dementia. *P_FDR_ < 0.05. Individual data points (circles) correspond to values from single subjects.

Within the composite frontotemporal ROI, both volume and autocorrelation-based texture features were statistically significant between bvFTD patients with mild dementia and HCs (*P* < 0.05, Table 2, Fig. 2).

**Table 2:** Group comparison of neuroimaging measures between HCs, bvFTD patients with mild dementia, and bvFTD patients with moderate dementia within the frontal-temporal region. Significant *P*-values after correction are bolded in table (*P*_FDR_ < 0.05)

Measure	Group				95% confidence interval	
	I	J	I—J	*P*	LB	UB
**Volume** cm^3^	HC	Mild	19	**1E-06**	11.0	27.6
	HC	Moderate	30	**6E-10**	20.7	39.9
	Mild	Moderate	11	0.03	0.6	21.3
**Autocorrelation** a. u	HC	Mild	29	**7E-04**	11.2	47.8
	HC	Moderate	56	**9E-08**	34.8	77.3
	Mild	Moderate	26	**0.01**	3.6	49.4

#### Frontotemporal subregions

**Figure 3:**
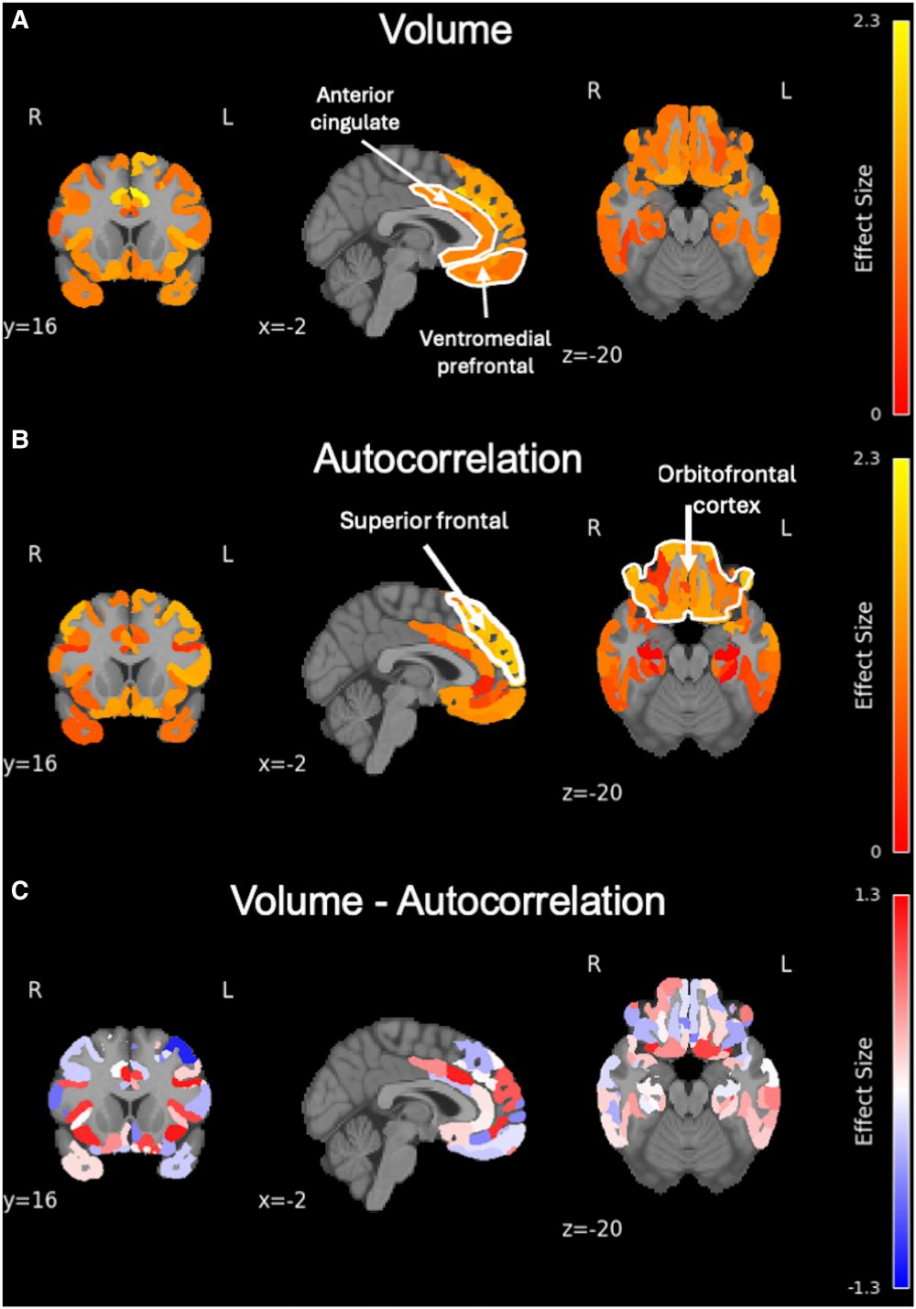
**Regional effect sizes from ANCOVA, adjusted for age and sex, comparing brain volume and texture between HCs (*n* = 33) and bvFTD patients with mild dementia (*n* = 21) across different brain slices.** (**A**) effect size values for group differences in brain volume across 160 subregions within the composite frontotemporal regions of interest, as defined by the Glasser segmentation. (**B**) effect size values for group differences in texture across the same 160 subregions. (**C**) Differences between effect size values for group differences in volume and texture across the same 160 subregions.

To explore specific regional differences in brain volume and texture, we analysed 160 subregions within the composite frontotemporal ROI, as defined by the Glasser segmentation. Among these, 158 regions showed significant differences in brain volume (Fig. 3A, *P_FDR_* < 0.05), and 115 regions showed significant differences in texture (Fig. 3B, *P_FDR_* < 0.05) between HCs and bvFTD patients with mild dementia. Regions demonstrating the largest effect sizes for brain volume were concentrated in the anterior cingulate and medial prefrontal cortices, whereas regions with the larges effect sizes for texture occurred in lateral orbitofrontal and inferior frontal cortices (Fig. 3C, Supplementary Tables 1 and 2).

### Group differences in brain volume and texture between bvFTD with mild and moderate dementia

#### Composite frontotemporal ROI

Within the composite frontotemporal ROI, only the autocorrelation-based texture feature was significantly different between bvFTD patients with mild dementia and bvFTD patients with moderate dementia (*P_FDR_*  *<* 0.05), (Fig. 2; Table 2**)**.

#### Frontotemporal subregions

**Figure 4:**
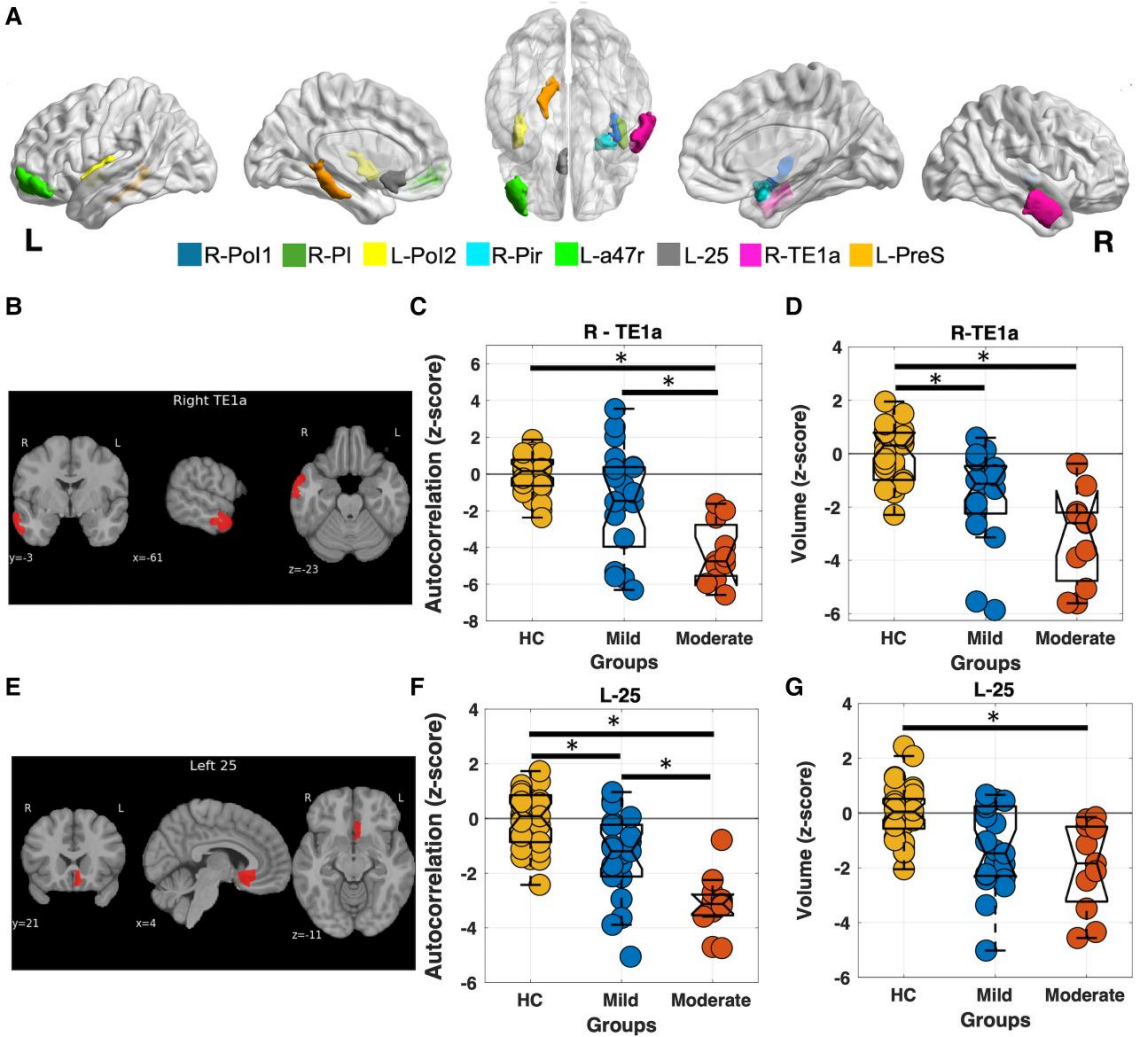
**Regional differences in brain volume and texture between bvFTD patients with mild (*n* = 21) and moderate dementia (*n* = 11).** (**A**) Brain regions showing significant group differences in texture. (B–D) Right TE1a: (**B**) region location, (**C**): autocorrelation texture differences [*P* = 0.002, 95% CI (20 104)], and (**D**): volume differences [*P* = 0.01, 95% CI (17 234)]. (E-G) Left 25: (**E**) region location, (**F**): autocorrelation texture differences [*P* = 0.002, 95% CI (15 80)] and (**G**): volume [*P* = 0.58, 95% CI (−48 115)]. Statistical comparisons were corrected for multiple testing (*P_FDR_* < 0.05, ANCOVA adjusted for age and sex). Individual data points (circles) correspond to values from single subjects. L: left, R: right, PreS: pre subiculum, PI: para insular area, a47r: area anterior 47r, PoI1: posterior insular area 1, Pir: piriform cortex, TE1a: area TE1 anterior, PoI2: posterior insular area 2, 25: area 25. *P_FDR_ < 0.05.

**Figure 5:**
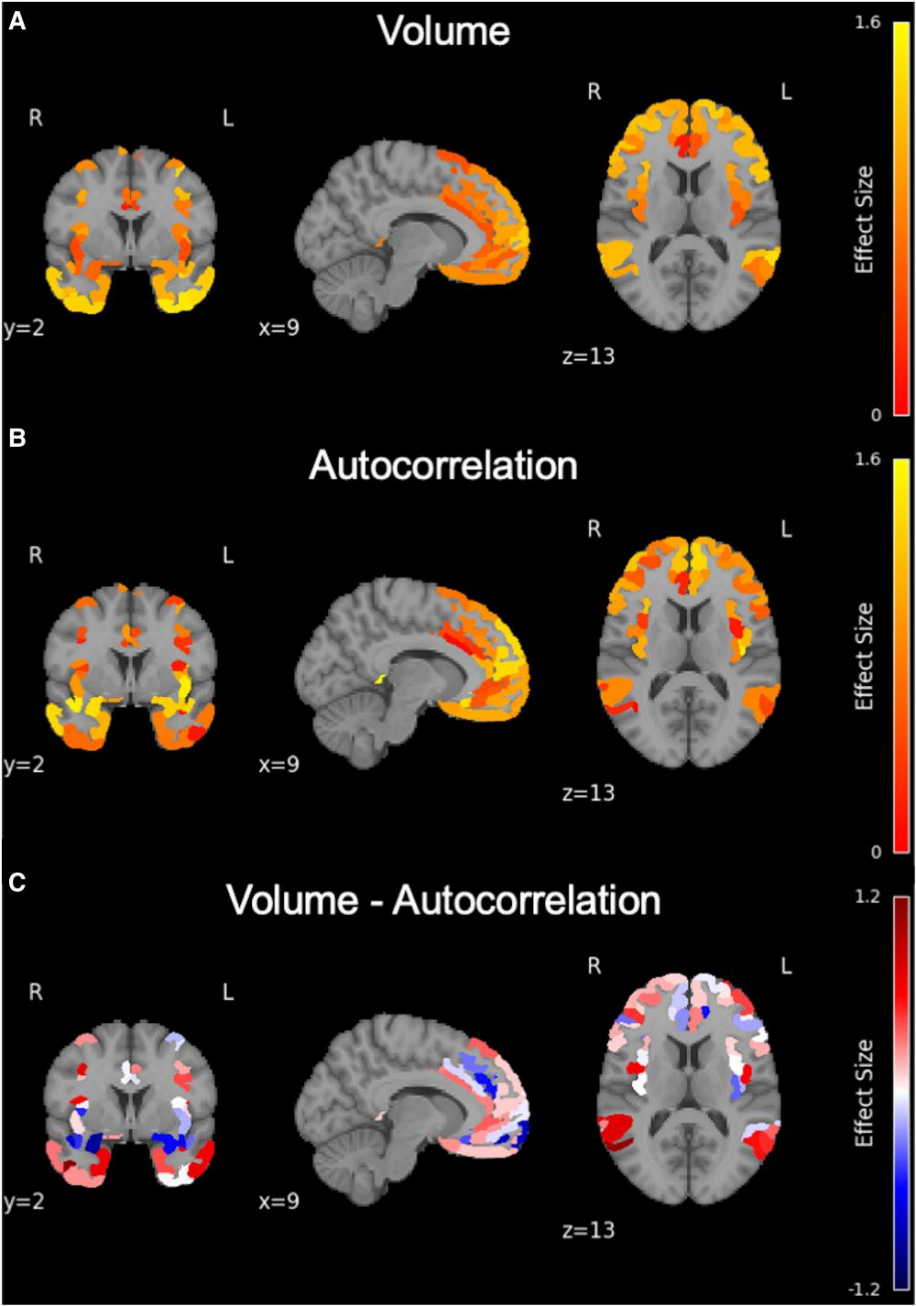
**Regional effect sizes from ANCOVA, adjusted for age and sex, comparing brain volume and texture between bvFTD patients with mild dementia (*n* = 21) and those with moderate dementia (*n* = 11) across different brain slices.** (**A**) Effect size values for group differences in brain volume across 160 subregions within the composite frontotemporal regions of interest, as defined by the Glasser segmentation. (**B**) Effect size values for group differences in texture across the same 160 subregions. (**C**) Differences between effect size values for group differences in volume and texture across the same 160 subregions.

No statistically significant differences in brain volume were observed between bvFTD patients with mild dementia and those with moderate dementia across these sub-ROIs (*P_FDR_* > 0.05). However, significant differences were observed in the autocorrelation-based texture feature. Specifically, bvFTD patients with moderate dementia showed significantly lower autocorrelation values in several subregions, including left pre subiculum (PreS), right para insular area (PI), left area anterior 47r (a47r), right area posterior insular 1 (PoI1), right piriform cortex (Pir), right area TE1 anterior (TE1a), left posterior insular area 2 (PoI2), left area 25 (25), compared to patients with mild dementia **(**Fig. 4, Table 3, Supplementary Fig. 3). To further indicate the relative contributions of volume and texture as progression markers, we plotted the effect sizes across all brain regions (Fig. 5). Figure 5A shows the effect sizes derived from volume, Fig. 5B shows effect sizes derived from autocorrelation-based texture, and Fig. 5C shows the difference between volume and texture effect sizes (volume—autocorrelation).

**Table 3:** Group comparison of neuroimaging measures between HC, bvFTD patients with mild dementia and bvFTD patients with moderate dementia within frontal and temporal subregions

ROI	Group		Volume results				Autocorrelation-based texture results			
					95% confidence interval				95% confidence interval	
	i	j	I—J	P*	LB	UB	I—J	P*	LB	UB
L-PreS Medial temporal	HC	Mild	35	8E−3	7.9	63.0	38	4E−3	10.5	67.2
	HC	Moderate	57	E−4	26.0	89.8	99	2E−09	66.8	132.5
	Mild	Moderate	22	0.26	−11.9	56.8	60	**3E**−**4**	25.3	96.2
R-PI Insular and frontal opercular	HC	Mild	37	2E−4	15.6	58.4	39	3E−3	11.6	67.8
	HC	Moderate	59	1E−06	34.3	83.8	96	4E−09	64.1	129.2
	Mild	Moderate	22	0.12	−4.6	48.7	56	**7E**−**4**	21.8	92.0
L-a47r Inferior frontal	HC	Mild	148	7E−05	70.4	226.6	82	1E−06	47.0	117.2
	HC	Moderate	242	6.E−08	151.8	332.8	150	4E−12	110.1	191.4
	Mild	Moderate	93	0.06	−3.7	191.4	68	**1E**−**3**	24.8	112.5
R-PoI1 Insular and frontal opercular	HC	Mild	82	6E−4	31.8	132.6	34	0.02	3.7	64.9
	HC	Moderate	108	1E−4	49.8	166.6	93	1E−07	57.9	128.8
	Mild	Moderate	25	0.58	−36.9	88.9	59	**1E**−**3**	20.8	97.3
R-Pir Insular and frontal opercular	HC	Mild	90	8E−4	34.0	147.8	31	0.09	−4.3	67.9
	HC	Moderate	128	5E−05	62.1	193.9	98	1E−06	56.7	140.5
	Mild	Moderate	37	0.42	−34.0	108.1	66	**2E**−**3**	21.6	112.0
R-TE1a Lateral temporal	HC	Mild	160	E−4	74.1	247.2	44	E−3	11.1	78.7
	HC	Moderate	286	1E−08	186.5	387.0	107	3E−08	68.2	146.5
	Mild	Moderate	126	0.01	17.9	234.2	62	**2E**−**3**	20.2	104.7
L-PoI2 Insular and frontal opercular	HC	Mild	115	7E−4	33.2	44.0	66	3E−05	33.2	99.4
	HC	Moderate	148	1E−4	186.7	65.9	127	1E−10	89.0	165.7
	Mild	Moderate	33	0.64	−55.8	122.4	61	**2E**−**3**	19.7	102.4
L-25 Anterior cingulate and medial prefrontal	HC	Mild	109	4E−04	44.2	175.7	49	8E−05	23.0	75.2
	HC	Moderate	143	8E−05	67.5	219.9	96	4E−10	66.6	127.1
	Mild	Moderate	33	0.58	−48.3	115.9	47	**2E**−**3**	15.1	80.3

*P*-values highlighted in bold are statistically significant between mild and moderate bvFTD patients after FDR correction (*P*_FDR_ < 0.05).

PreS = PreSubiculum, PI = Para-Insular_Area, a47r = Area_anterior_47r, PoI1 = Area_Posterior_Insular_1, Pir = Pirform_Cortex, TE1a = Area_TE1_anterior, PoI2 = Posterior_Insular_Area_2, 25 = Area_25, L = Left, R = Right.

^*^ *P*-values are uncorrected.

#### Comparison between volume and autocorrelation-based texture feature models

There was no statistical difference between volume and autocorrelation-based texture feature in distinguishing between HCs versus patients with mild dementia (volume AUC = 0.84, autocorrelation AUC = 0.85, volume + autocorrelation AUC = 0.87, *P* = 0.33), or between patients with mild dementia versus patients with moderate dementia (volume AUC = 0.63, autocorrelation AUC = 0.64, volume + autocorrelation AUC = 0.63, *P* = 1) (Supplementary Fig. 4). Combining volume and texture marginally improved classification between HCs versus patients with mild dementia, although this improvement was not statistically significant from models with each measure individually.

#### The relationship between texture feature and volume

A significant positive linear relationship was observed between brain volume and the autocorrelation-based texture feature within the composite frontotemporal region (*P* = 1 × 10⁻⁵, *β* = 0.001). However, when group was included as a covariate, this relationship was no longer significant (*P* = 0.15, *β* = 0.0004). Moreover, there was no linear relationship between texture and volume in either HC (*P* = 0.15, *β* = 0.00004) or bvFTD patients (*P* = 0.16, *β* = 0.0004) when the groups were evaluated separately.

At the regional level, a significant positive linear association between volume and the autocorrelation-based texture feature was found in 125 out of 160 ROIs (*P_FDR_* < 0.05; Supplementary Table 3, Supplementary Fig. 5A). After adjusting for group, this relationship remained significant in only 33 out of 160 ROIs (*P_FDR_* < 0.05; Supplementary Table 3, Supplementary Fig. 5B). When we are looking at the brain regions across each group separately, the only significant finding was a negative relationship between volume and texture within left para hippocampal area 2 in the healthy group (*P* = 0.0002, *β* = −1.28).

## Discussion

In this study, we demonstrate that microstructural abnormalities, as measured by autocorrelation-based texture features, can distinguish bvFTD patients with mild dementia from HCs, with increased sensitivity to changes within orbitofrontal, inferior frontal and dorsomedial/superior frontal regions compared to volumetric measures. We further demonstrate that microstructural abnormalities can differentiate bvFTD patients with mild versus moderate dementia severity, even in the absence of detectable volumetric changes, specifically within orbitofrontal, insula, anterior cingulate and anterior temporal regions. These results highlight the potential of microstructural alterations, detectable through texture analysis, as promising candidate metrices for assessing bvFTD severity.

### Early detection: differentiating healthy controls from bvFTD patients with mild dementia

One single prior study evaluated texture analysis in bvFTD, finding that textural features could discriminate bvFTD from HCs and other types of FTLD.^30^ In Alzheimer’s disease, hippocampal textural features have been shown to discriminate Alzheimer’s disease from HCs, or semantic variant primary progressive aphasia.^24,26,28,31^ Moreover, texture but not volumetric measures discriminated patients with Alzheimer’s disease versus svPPA.^31^ However, to our knowledge, our study is the first to compare texture and volumetric analysis and the first to demonstrate detection of microstructural differences specifically in bvFTD patients.

Our findings indicate that both microstructural abnormalities in grey matter and regional volumetric changes are different between bvFTD patients with mild dementia versus HCs across multiple regions within the frontotemporal lobes (Figs 2 and 3). Consistent with previous studies, our volumetric analysis shows that atrophy is bilateral.^44^

Our results indicate that the most significantly affected brain regions in terms of volume are primarily located within the anterior cingulate and medial prefrontal cortices (Fig. 3A, Supplementary Table 1), consistent with patterns of atrophy commonly observed in bvFTD.^45-48^ In contrast, the regions showing the most significant differences in the autocorrelation-based texture feature were located in orbitofrontal, inferior frontal and dorsomedial/superior frontal regions (Fig. 3B, Supplementary Table 2), suggesting that texture features may capture complementary or distinct aspects of neurodegeneration compared to volumetric analysis, highlighting their potential utility as a complementary measure for detecting early-stage neurodegeneration in bvFTD.

Furthermore, ROC analysis within the composite frontotemporal ROI demonstrates that both volume and autocorrelation exhibit similar performance (*P_DeLong_* > 0.05). Volume shows a sensitivity of 0.76, and a specificity of 0.84, while autocorrelation shows a sensitivity of 0.81 and a specificity of 0.84 (Supplementary Fig. 4). Prior imaging-based classification approaches using morphometric or ventricular measures have reported comparable accuracies (typically 0.72–0.89) in distinguishing bvFTD from HCs and other clinical groups.^49-53^

An important difference between our study and these other studies is that we focused on differentiating patients with mild bvFTD versus HCs, whereas other studies included bvFTD patients with greater disease severity, which likely increased the sensitivity and specificity for their classifications in comparison to our results.

### Disease progression: bvFTD patients with mild versus moderate dementia

We found that microstructural abnormalities in grey matter were more pronounced in bvFTD patients with moderate dementia compared to those with mild dementia, even in the absence of detectable reductions in brain volume (Tables 2 and 3, Figs 2 and 4). The higher performance of autocorrelation compared to volume within specific regions suggests that texture features are more sensitive to neurodegeneration from mild to moderate dementia, making texture-based measures potentially useful neuroimaging biomarkers for tracking disease progression. Moreover, most regions exhibit similar effect sizes: specific regions demonstrate larger effect sizes for one metric over the other, highlighting that texture analysis can provide complementary information to volumetric measures (Fig. 5).

Texture features have also tracked with disease stage and progression in Alzheimer’s disease. For example, texture features derived from the hippocampus can distinguish Alzheimer’s disease from MCI.^24,26,28,31^ Moreover, texture measures also better predicted conversion from MCI to Alzheimer’s disease ^54,55^ and better correlated with cognitive measures than volume in patients with Alzheimer’s disease.^24^ To our knowledge, this is the first neuroimaging study to identify stage-specific microstructural atrophy patterns in bvFTD.

Additionally, we observed higher variability in the autocorrelation-based texture feature among patients with mild dementia compared with volume, suggesting texture captures a broader spectrum of individual differences. In contrast, patients with moderate dementia exhibited less variability, indicating a more homogeneous profile. Specifically, autocorrelation within the mild dementia group appeared to dichotomize patients into those with values similar to the moderate dementia group, versus those with milder differences. This suggests that while autocorrelation-based texture features are sensitive to subtle, individualized changes early in the disease, they become more consistent in later stages of the disease. It also remains unknown whether mild dementia patients with more advanced texture features might be at greater risk for progression to moderate dementia, hypotheses that could be testing in future studies with longitudinal assessments.

### Interpretation of texture and volume measures of neurodegeneration in bvFTD

Our findings indicate considerable overlap between regions showing microstructural abnormalities, as measured by texture analysis, and those exhibiting volume loss in bvFTD patients with mild dementia compared to HCs (Fig. 3). However, the magnitude of group differences varies across regions, with some areas showing more pronounced changes in volume, and others showing greater differences in texture. This suggests that although both markers capture disease-related alterations, they may reflect distinct or complementary aspects of the underlying pathology.

The regional variability in the strength of texture and volume differences may reflect differences in the temporal sequence of disease progression or the specific nature of tissue damage. In some brain regions, microstructural changes captured by texture features may emerge earlier and precede detectable atrophy, indicating early degeneration. In contrast, other regions may undergo more rapid or overt volume loss, possibly reflecting more advanced or macrostructural damage. These patterns may also be shaped by region-specific susceptibility to different pathological proteins (e.g. tau, TDP-43) or distinct pathways of disease spread across brain networks. Further longitudinal studies are needed to better understand the mechanisms driving these relationships and to evaluate whether texture features can serve as earlier or more sensitive markers of neurodegeneration.

Furthermore, we observed that while many frontotemporal brain regions show significant differences in both texture and volume between HCs and individuals with mild dementia, only texture differences persist when comparing individuals with mild versus moderate dementia (Fig. 4, Table 3, Supplementary Fig. 3). This suggests that in these regions, microstructural changes continue to progress with disease severity, even when volume remains relatively stable. These findings highlight the greater sensitivity of texture features in detecting subtle, ongoing neurodegenerative changes that may not yet be reflected in gross volumetric atrophy. As such, texture analysis may provide complementary or earlier indicators of disease progression beyond what is captured by traditional volume-based measures.

Finally, our results reveal subregions where only volumetric differences are observed between HCs and individuals with mild dementia, with no further changes in volume between mild and moderate dementia (42 ROIs). Two hypotheses may explain these findings. First, these regions could experience significant structural damage early in the disease course, leading to a plateau where further progression does not cause additional volumetric loss. Alternatively, these regions may only sustain mild initial damage, which remains stable over time without further degeneration.

### The relationship between texture and volume

We found a significant positive linear relationship between brain volume and the autocorrelation-based texture feature across our combined sample, but much weaker associations after including group as a covariate (Supplementary Fig. 5). This substantial reduction in significant associations after accounting for group suggests that the initial strong relationship between volume and texture was largely driven by between-group differences (the co-occurrence of volumetric and texture alterations in bvFTD patients compared to controls) rather than within-group variation. However, in the 33 ROIs where the relationship remained significant even after adjusting for group, texture may provide complementary, independent or more sensitive information related to neurodegeneration than volumetric measures alone.

To our knowledge, although many prior studies have collected both texture and volume measures, they have not directly assessed the association between these features within specific ROIs. Combined with our finding that patients with bvFTD exhibited reduced texture but no detectable volume loss in certain regions, these results suggest that texture features may be particularly sensitive to subtle neurodegenerative changes across different disease stages.

While speculative, early neurodegenerative changes detected by texture analysis could represent deposition of neuropathological proteins (e.g. tau and TDP-43). In Alzheimer’s disease, textural features correlate with tau but not beta amyloid deposition using PET radiotracers, supporting this potential interpretation.^29^ Alternatively, changes to texture could reflect early synaptic changes that result from abnormal protein deposition prior to neuronal cell death. Supporting this hypothesis, prior studies have found that texture changes correlated with glucose metabolism measures using FDG-PET within the hippocampus and with functional connectivity between the hippocampus and other regions in patients with Alzheimer’s disease.^24,56^ Third, textural changes could reflect inflammation triggered by neuropathological protein deposition, supported by studies linking hippocampal texture to blood-based markers of myeloid leukocyte and neutrophil activation in patients with Alzheimer’s disease.^57^ Finally, texture features could reflect neuronal death that is not extensive enough to result in obvious volumetric changes (Supplementary Fig. 6).

### Limitations

This study has several limitations. First, our findings are based on a relatively small sample size from a single centre. While a single-centre design is common in bvFTD research and offers the advantage of consistent and rigorous protocols to reduce variability and confounders, this may restrict the generalizability of our results. Second, like most bvFTD studies, our analysis is cross-sectional, and key hypotheses regarding the ability for texture to track disease progression are being actively pursued in longitudinal follow-up analyses. Finally, while prior studies have associated changes in texture analysis with neuropathology, inflammation, and functional neuroimaging changes in Alzheimer’s disease, similar investigations are necessary in FTD. This study offers an important initial step by demonstrating texture abnormalities in FTD, establishing a basis for future studies aimed at linking these imaging features to underlying molecular and histopathological mechanisms.

## Conclusions

Microstructural abnormalities identified using texture analysis occur in patients with mild bvFTD and can differentiate bvFTD patients with mild versus moderate dementia severity even in the absence of volumetric changes, suggesting that texture-based MRI metrics may complement traditional volumetric MRI for clinical practice. While these findings may not translate into immediate diagnostic changes, they demonstrate that clinically available T1-weighted MRI can reveal microstructural changes that evolve across disease stages, providing a more nuanced picture of neurodegeneration. Because the analysis is based on standard T1-weighted MRI, which is already routinely acquired in patients, these methods could be readily integrated into existing workflows to extract additional clinically relevant information at no extra cost or burden. This approach could help clinicians identify patients at earlier stages of disease, refine prognostic evaluations and make more informed referrals for therapeutic trials. Furthermore, by demonstrating sensitivity to disease progression, our study underscores the importance of longitudinal imaging for tracking neurodegeneration and evaluating treatment efficacy, positioning texture analysis as a promising tool both for patient care and for advancing clinical research.

## Supplementary Material

fcaf427_Supplementary_Data

## Data Availability

The data that support the findings of this study are available from the corresponding author upon reasonable request. All scripts and codes are publicly available at https://github.com/Behnaz-Akbarian/bvFTD-texture-analysis_paper.
