# Supplementary material for: Microstructural grey matter alterations in patients with behavioural variant frontotemporal dementia
Source: Brain Commun. 2025 Nov 3;7(6):fcaf427. doi: 10.1093/braincomms/fcaf427 (PMC12609174; doi:10.1093/braincomms/fcaf427)
Supplement: fcaf427_Supplementary_Data [file fcaf427_supplementary_data.docx]

**Microstructural gray matter alterations in patients with behavioral variant frontotemporal dementia**

**Supplementary material**

**
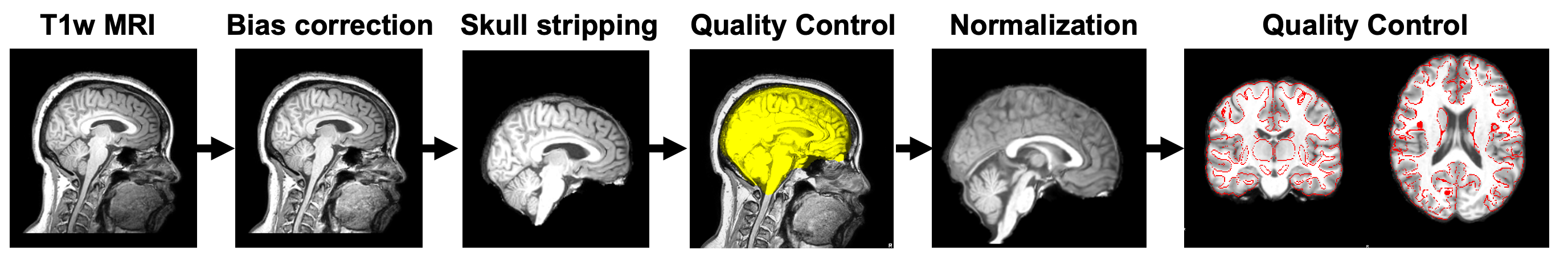
**

**Supplementary Figure 1. Preprocessing pipeline for texture analysis of T1-weighted MRI images**. The pipeline includes (1) bias field correction using the N4 method, (2) skull stripping with FreeSurfer’s recon, followed by a quality control (QC) check to ensure accurate brain extraction, and (3) normalization to the Glasser atlas using Advanced Normalization Tools (ANTs), with an additional QC step to verify accurate segmentation.


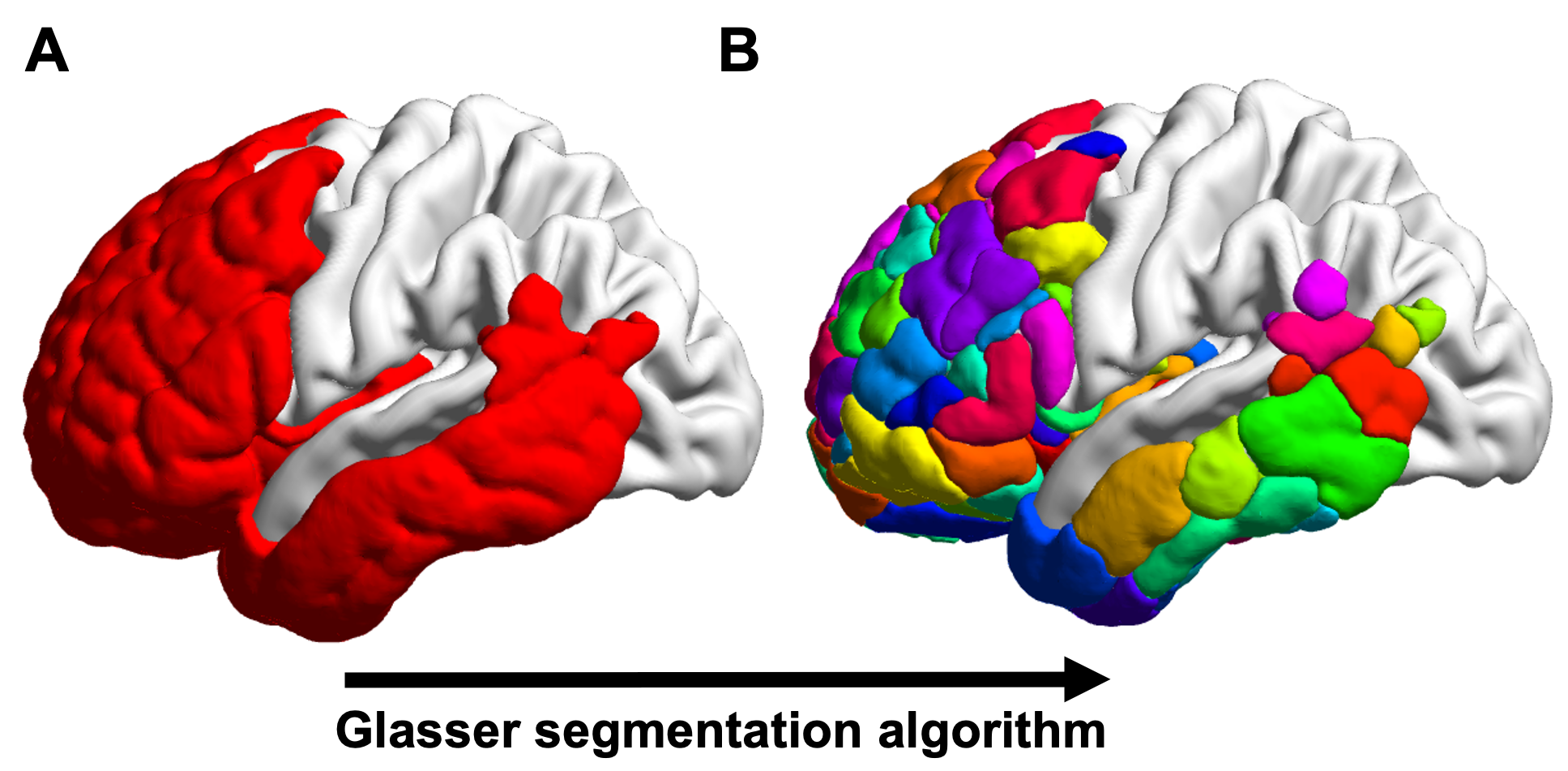


**Supplementary Figure 2.** **Regions of interest (ROIs) selected for analysis**. (A) Composite frontotemporal ROI. And (B) 160 frontotemporal subregions within the frontal and temporal lobes, segmented using the Glasser Atlas.

**Supplementary Table 1.** The first ten ROIs with significant volume differences between behavior variant frontotemporal dementia (bvFTD) patients with mild dementia and healthy controls.

| ID | Region | Region Long Name | Cortical Division | Cortex |
| --- | --- | --- | --- | --- |
| 142 | 'L_a32pr' | 'Area_anterior_32_prime' | 'Anterior_Cingulate_and_Medial_Prefrontal' | 19 |
| 147 | 'L_p32pr' | 'Area_p32_prime' | 'Anterior_Cingulate_and_Medial_Prefrontal' | 19 |
| 322 | 'R_a32pr' | 'Area_anterior_32_prime' | 'Anterior_Cingulate_and_Medial_Prefrontal' | 19 |
| 327 | 'R_p32pr' | 'Area_p32_prime' | 'Anterior_Cingulate_and_Medial_Prefrontal' | 19 |
| 144 | 'L_p24' | 'Area_posterior_24' | 'Anterior_Cingulate_and_Medial_Prefrontal' | 19 |
| 323 | 'R_d32' | 'Area_dorsal_32' | 'Anterior_Cingulate_and_Medial_Prefrontal' | 19 |
| 143 | 'L_d32' | 'Area_dorsal_32' | 'Anterior_Cingulate_and_Medial_Prefrontal' | 19 |
| 253 | 'R_FOP5' | 'Area_Frontal_Opercular_5' | 'Insular_and_Frontal_Opercular' | 12 |
| 326 | 'R_p32' | 'Area_p32' | 'Anterior_Cingulate_and_Medial_Prefrontal' | 19 |
| 319 | 'R_9m' | 'Area_9_Middle' | 'Anterior_Cingulate_and_Medial_Prefrontal' | 19 |

**Supplementary Table 2.** The first ten ROIs with significant texture differences between bvFTD patients with mild dementia and healthy controls.

| ID | Region | Region Long Name | Cortical Division | Cortex |
| --- | --- | --- | --- | --- |
| 139 | 'L_9m' | 'Area_9_Middle' | 'Anterior_Cingulate_and_Medial_Prefrontal' | 19 |
| 171 | 'L_8BL' | 'Area_8B_Lateral' | 'Dorsolateral_Prefrontal' | 22 |
| 340 | 'R_45' | 'Area_45' | 'Inferior_Frontal' | 21 |
| 161 | 'L_47l' | 'Area_47l_(47_lateral)' | 'Inferior_Frontal' | 21 |
| 342 | 'R_a47r' | 'Area_anterior_47r' | 'Inferior_Frontal' | 21 |
| 356 | 'R_a9-46v' | 'Area_anterior_9-46v' | 'Dorsolateral_Prefrontal' | 22 |
| 319 | 'R_9m' | 'Area_9_Middle' | 'Anterior_Cingulate_and_Medial_Prefrontal' | 19 |
| 72 | 'L_FOP4' | 'Frontal_Opercular_Area_4' | 'Insular_and_Frontal_Opercular' | 12 |
| 323 | 'R_d32' | 'Area_dorsal_32' | 'Anterior_Cingulate_and_Medial_Prefrontal' | 19 |
| 162 | 'L_a47r' | 'Area_anterior_47r' | 'Inferior_Frontal' | 21 |


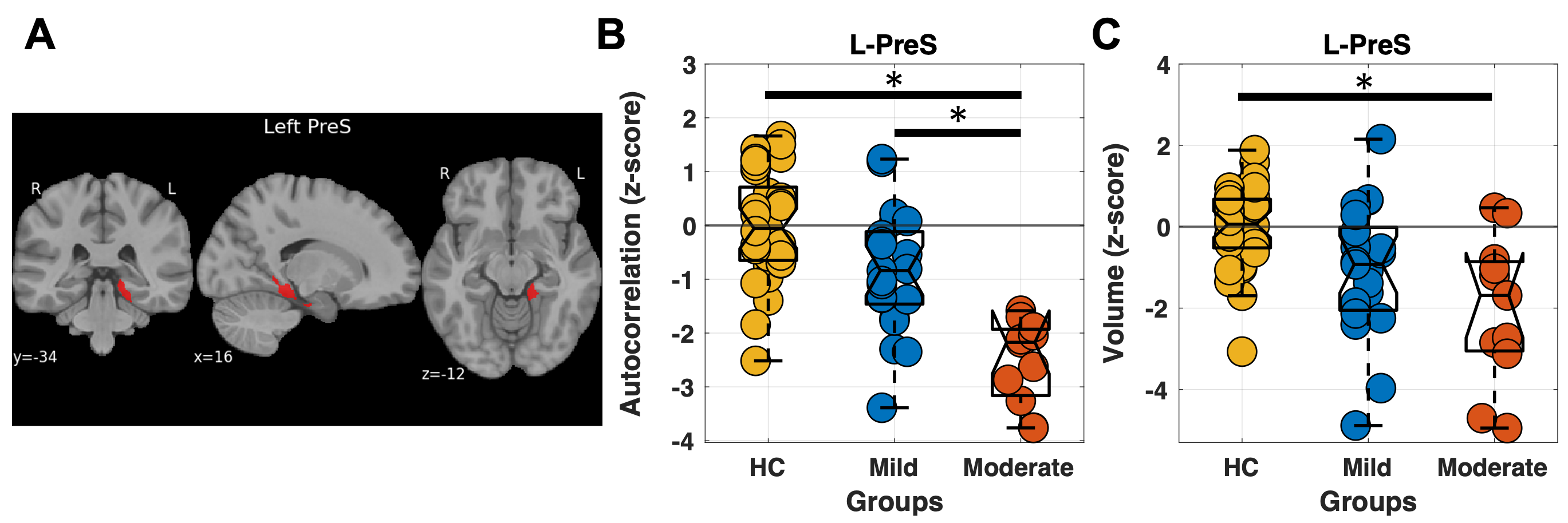

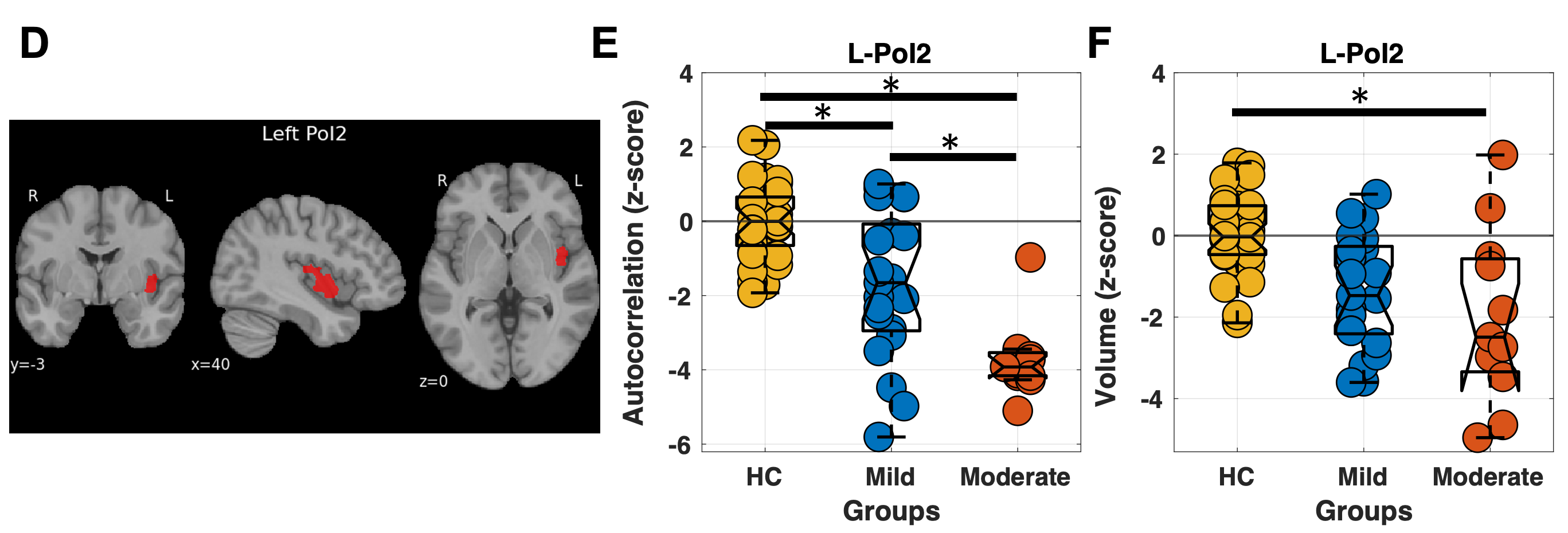

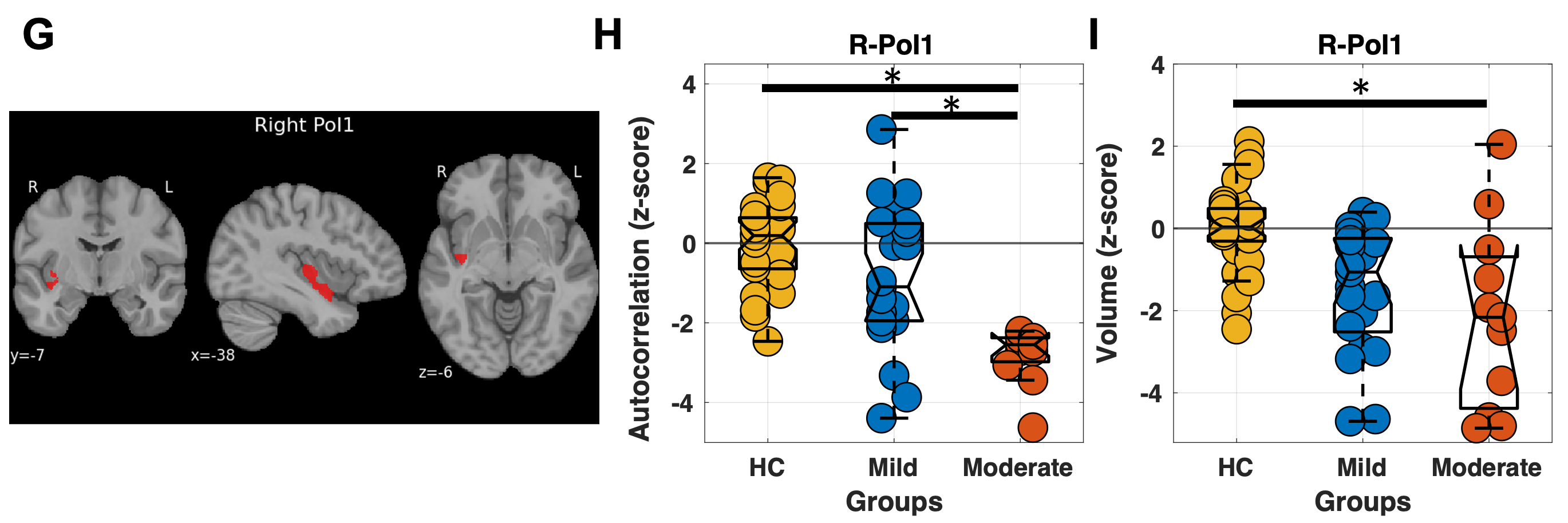

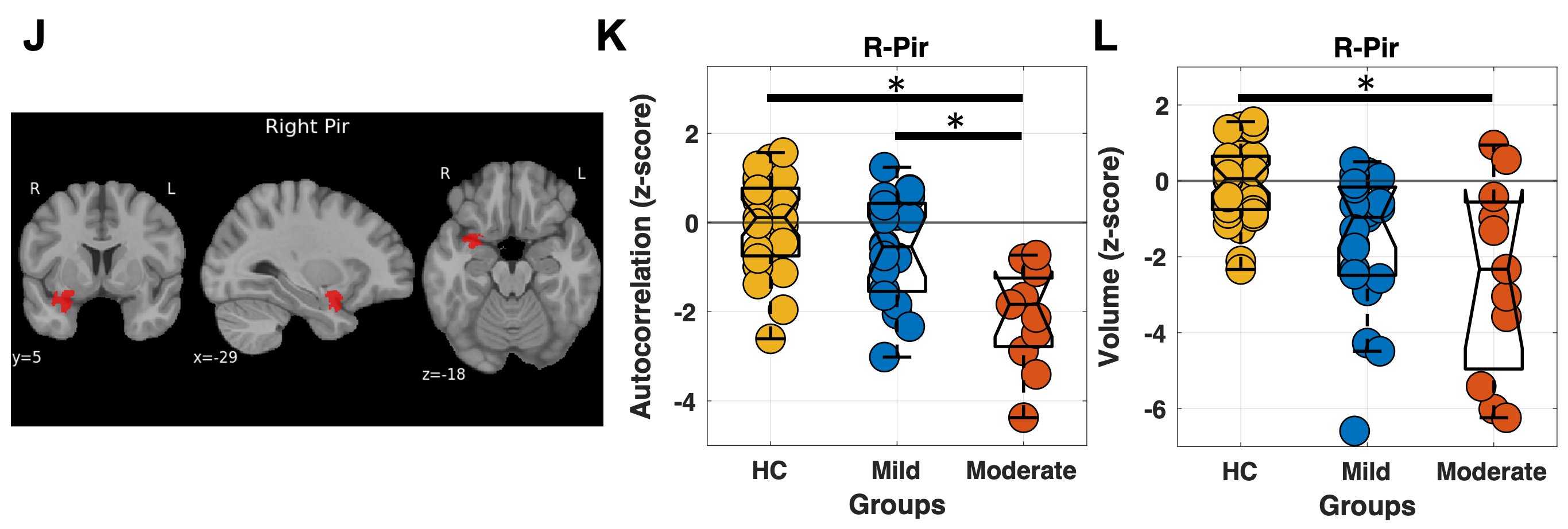

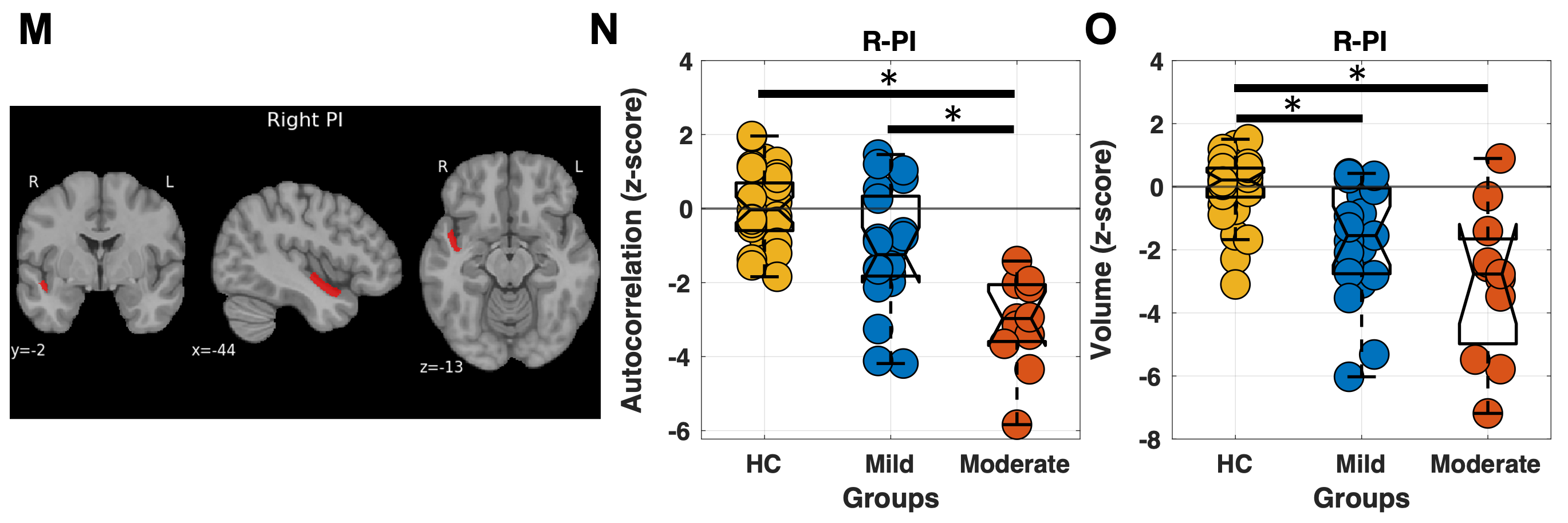

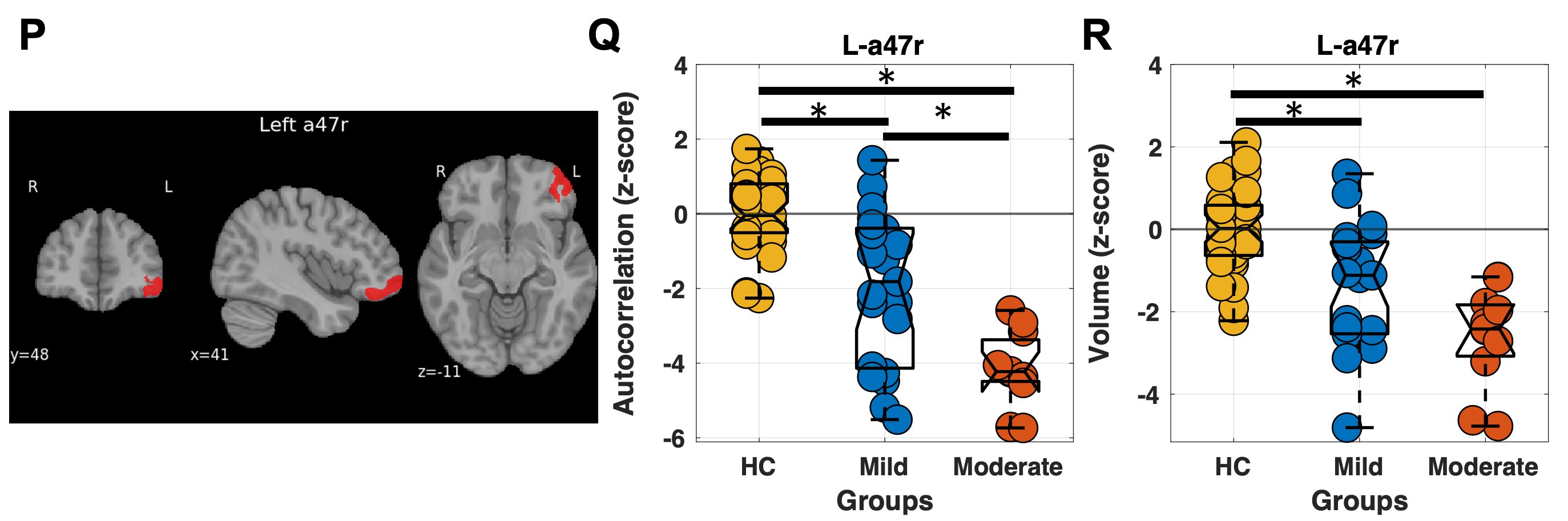


**Supplementary Figure 3. Regional differences in brain volume and texture between behavioral variant frontotemporal dementia (bvFTD) patients with mild (n = 21), and moderate dementia (n = 11)**. Within (A-C) left pre subiculum (PreS): (A) region location, (B): autocorrelation texture differences (P = 0.0003, 95% CI [25 96]), and (C): volume differences (P = 0.26, 95% CI [-11 56]), (D-F) left posterior insular area 2 (PoI2): (D) region location, (E): autocorrelation texture differences (P = 0.0002, 95% CI [19 102]), and (F): volume differences (P = 0.64, 95% CI [-55 122]), (G-I) right area posterior insular 1 (PoI1): (G) region location, (H): autocorrelation texture differences (P = 0.001, 95% CI [20 97]), and (I): volume differences (P = 0.58, 95% CI [-36 88]), (J-L) right piriform cortex (Pir): (J) region location, (K): autocorrelation texture differences (P = 0.002, 95% CI [21 112]), and (L): volume differences (P = 0.42, 95% CI [-34 108]), (M-O) right para insular area (PI): (M) region location, (N): autocorrelation texture differences (P = 0.0007, 95% CI [21 92]), and (O): volume differences (P = 0.12, 95% CI [-4 48]), and (P-R) left area anterior 47r (a47r): (P) region location, (Q): autocorrelation texture differences (P = 0.001, 95% CI [24 112]), and (R): volume differences (P = 0.06, 95% CI [-3 191]). ANCOVA adjusted for age and sex. (*: *P_FDR_* < 0.05). Individual data points (circles) correspond to values from single subjects.


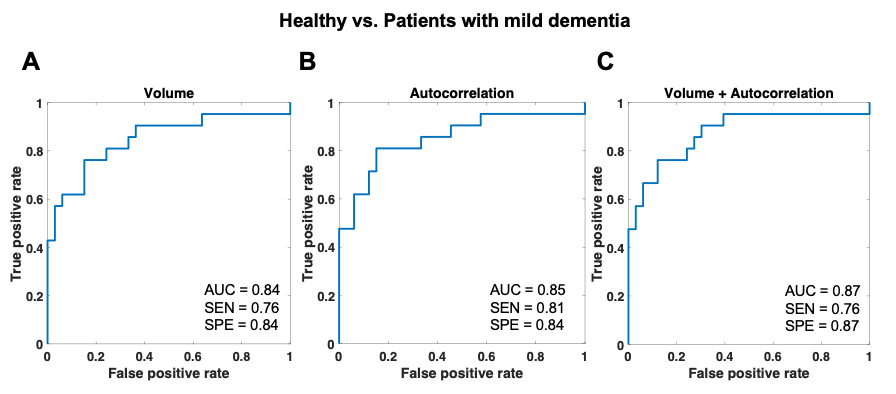


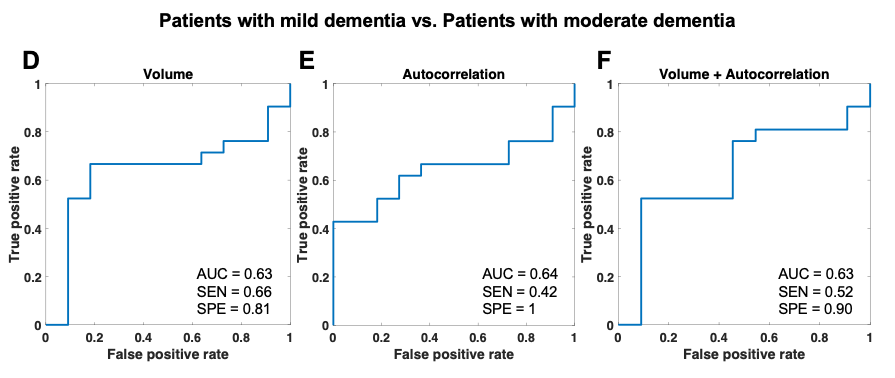


**Supplementary Figure 4.** **Receiver operating characteristic (ROC) analysis**. ROC for volume, autocorrelation and combination of volume and autocorrelation within composite frontotemporal region of interest respectively: for **(**A-C**)** healthy controls (n = 33) vs. behavioral variant frontotemporal dementia (bvFTD) patients with mild dementia (n = 21), and **(**D-F**)** bvFTD patients with mild vs. moderate dementia (n = 22). AUC: area under curve, SEN: sensitivity, SPE: specificity.

**Supplementary Table 3.** ROIs showing a significant positive linear relationship between volume and the autocorrelation-based texture feature (125 ROIs, *P_FDR_* < 0.05) without adjusting for group. Highlighted regions remain significant after adjusting for group.

| ID | Region | RegionLongName | Cortical Division | Cortex |
| --- | --- | --- | --- | --- |
| 68, 248 | 'AAIC' | 'Anterior_Agranular_Insula_Complex' | 'Insular_and_Frontal_Opercular' | 12 |
| 72, 252 | 'FOP4' | 'Frontal_Opercular_Area_4' | 'Insular_and_Frontal_Opercular' | 12 |
| 73, 253 | 'FOP5' | 'Area_Frontal_Opercular_5' | 'Insular_and_Frontal_Opercular' | 12 |
| 74, 254 | 'Ig' | 'Insular_Granular_Complex' | 'Insular_and_Frontal_Opercular' | 12 |
| 75, 255 | 'MI' | 'Middle_Insular_Area' | 'Insular_and_Frontal_Opercular' | 12 |
| 76, 256 | 'PI' | 'Para-Insular_Area' | 'Insular_and_Frontal_Opercular' | 12 |
| 77 | 'L_Pir' | 'Pirform_Cortex' | 'Insular_and_Frontal_Opercular' | 12 |
| 78, 258 | 'PoI1' | 'Area_Posterior_Insular_1' | 'Insular_and_Frontal_Opercular' | 12 |
| 79, 259 | 'PoI2' | 'Posterior_Insular_Area_2' | 'Insular_and_Frontal_Opercular' | 12 |
| 81, 261 | 'PreS' | 'PreSubiculum' | 'Medial_Temporal' | 13 |
| 82, 262 | 'EC' | 'Entorhinal_Cortex' | 'Medial_Temporal' | 13 |
| 86, 266 | 'PHA2' | 'ParaHippocampal_Area_2' | 'Medial_Temporal' | 13 |
| 88, 268 | 'PHT' | 'Area_PHT' | 'Lateral_Temporal' | 14 |
| 89, 269 | 'TE1a' | 'Area_TE1_anterior' | 'Lateral_Temporal' | 14 |
| 90, 270 | 'TE1m' | 'Area_TE1_Middle' | 'Lateral_Temporal' | 14 |
| 91, 271 | 'TE1p' | 'Area_TE1_posterior' | 'Lateral_Temporal' | 14 |
| 92, 272 | 'TE2a' | 'Area_TE2_anterior' | 'Lateral_Temporal' | 14 |
| 94, 274 | 'TGd' | 'Area_TG_dorsal' | 'Lateral_Temporal' | 14 |
| 95, 275 | 'TGv' | 'Area_TG_Ventral' | 'Lateral_Temporal' | 14 |
| 96, 276 | 'PSL' | 'PeriSylvian_Language_Area' | 'Temporo-Parieto_Occipital_Junction' | 15 |
| 97 | 'L_STV' | 'Superior_Temporal_Visual_Area' | 'Temporo-Parieto-Occipital_Junction' | 15 |
| 98, 278 | 'TPOJ1' | 'Area_TemporoParietoOccipital_Junction_1' | 'Temporo-Parieto-Occipital_Junction' | 15 |
| 99 | 'L_TPOJ2' | 'Area_TemporoParietoOccipital_Junction_2' | 'Temporo-Parieto-Occipital_Junction' | 15 |
| 134 | 'L_10r' | 'Area_10r' | 'Anterior_Cingulate_and_Medial_Prefrontal' | 19 |
| 135, 315 | '10v' | 'Area_10v' | 'Anterior_Cingulate_and_Medial_Prefrontal' | 19 |
| 136, 316 | '25' | 'Area_25' | 'Anterior_Cingulate_and_Medial_Prefrontal' | 19 |
| 137 | 'L_33pr' | 'Area_33_prime' | 'Anterior_Cingulate_and_Medial_Prefrontal' | 19 |
| 138, 318 | '8BM' | 'Area_8BM' | 'Anterior_Cingulate_and_Medial_Prefrontal' | 19 |
| 139, 319 | '9m' | 'Area_9_Middle' | 'Anterior_Cingulate_and_Medial_Prefrontal' | 19 |
| 141, 321 | 'a24pr' | 'Anterior_24_prime' | 'Anterior_Cingulate_and_Medial_Prefrontal' | 19 |
| 142, 322 | 'a32pr' | 'Area_anterior_32_prime' | 'Anterior_Cingulate_and_Medial_Prefrontal' | 19 |
| 143, 323 | 'd32' | 'Area_dorsal_32' | 'Anterior_Cingulate_and_Medial_Prefrontal' | 19 |
| 144, 324 | 'p24' | 'Area_posterior_24' | 'Anterior_Cingulate_and_Medial_Prefrontal' | 19 |
| 145, 325 | 'p24pr' | 'Area_Posterior_24_prime' | 'Anterior_Cingulate_and_Medial_Prefrontal' | 19 |
| 146, 326 | 'p32' | 'Area_p32' | 'Anterior_Cingulate_and_Medial_Prefrontal' | 19 |
| 147, 327 | 'p32pr' | 'Area_p32_prime' | 'Anterior_Cingulate_and_Medial_Prefrontal' | 19 |
| 148, 328 | 'pOFC' | 'Posterior_OFC_Complex' | 'Anterior_Cingulate_and_Medial_Prefrontal' | 19 |
| 149 | 'L_s32' | 'Area_s32' | 'Anterior_Cingulate_and_Medial_Prefrontal' | 19 |
| 150, 330 | '10d' | 'Area_10d' | 'Orbital_and_Polar_Frontal' | 20 |
| 151, 331 | '10pp' | 'Polar_10p' | 'Orbital_and_Polar_Frontal' | 20 |
| 152 | 'L_11l' | 'Area_11l' | 'Orbital_and_Polar_Frontal' | 20 |
| 155, 335 | '47s' | 'Area_47s' | 'Orbital_and_Polar_Frontal' | 20 |
| 156, 336 | 'a10p' | 'Area_anterior_10p' | 'Orbital_and_Polar_Frontal' | 20 |
| 157, 337 | 'OFC' | 'Orbital_Frontal_Complex' | 'Orbital_and_Polar_Frontal' | 20 |
| 158, 338 | 'p10p' | 'Area_posterior_10p' | 'Orbital_and_Polar_Frontal' | 20 |
| 159, 339 | '44' | 'Area_44' | 'Inferior_Frontal' | 21 |
| 160, 340 | '45' | 'Area_45' | 'Inferior_Frontal' | 21 |
| 161, 341 | '47l' | 'Area_47l_(47_lateral)' | 'Inferior_Frontal' | 21 |
| 162, 342 | 'a47r' | 'Area_anterior_47r' | 'Inferior_Frontal' | 21 |
| 165, 345 | 'IFSa' | 'Area_IFSa' | 'Inferior_Frontal' | 21 |
| 166 | 'L_IFSp' | 'Area_IFSp' | 'Inferior_Frontal' | 21 |
| 167, 347 | 'p47r' | 'Area_posterior_47r' | 'Inferior_Frontal' | 21 |
| 168, 348 | '46' | 'Area_46' | 'Dorsolateral_Prefrontal' | 22 |
| 169, 349 | '8Ad' | 'Area_8Ad' | 'Dorsolateral_Prefrontal' | 22 |
| 170, 350 | '8Av' | 'Area_8Av' | 'Dorsolateral_Prefrontal' | 22 |
| 171, 351 | '8BL' | 'Area_8B_Lateral' | 'Dorsolateral_Prefrontal' | 22 |
| 172, 352 | '8C' | 'Area_8C' | 'Dorsolateral_Prefrontal' | 22 |
| 173, 353 | '9-46d' | 'Area_9-46d' | 'Dorsolateral_Prefrontal' | 22 |
| 174, 354 | '9a' | 'Area_9_anterior' | 'Dorsolateral_Prefrontal' | 22 |
| 175, 355 | '9p' | 'Area_9_Posterior' | 'Dorsolateral_Prefrontal' | 22 |
| 176, 356 | 'a9-46v' | 'Area_anterior_9-46v' | 'Dorsolateral_Prefrontal' | 22 |
| 177, 357 | 'i6-8' | 'Inferior_6-8_Transitional_Area' | 'Dorsolateral_Prefrontal' | 22 |
| 178, 358 | 'p9-46v' | 'Area_posterior_9-46v' | 'Dorsolateral_Prefrontal' | 22 |
| 179, 359 | 's6-8' | 'Superior_6-8_Transitional_Area' | 'Dorsolateral_Prefrontal' | 22 |
| 180, 360 | 'SFL' | 'Superior_Frontal_Language_Area' | 'Dorsolateral_Prefrontal' | 22 |
| 251 | 'R_FOP3' | 'Frontal_Opercular_Area_3' | 'Insular_and_Frontal_Opercular' | 12 |
| 263 | 'R_PeEc' | 'Perirhinal_Ectorhinal_Cortex' | 'Medial_Temporal' | 13 |
| 334 | 'R_47m' | 'Area_47m' | 'Orbital_and_Polar_Frontal' | 20 |


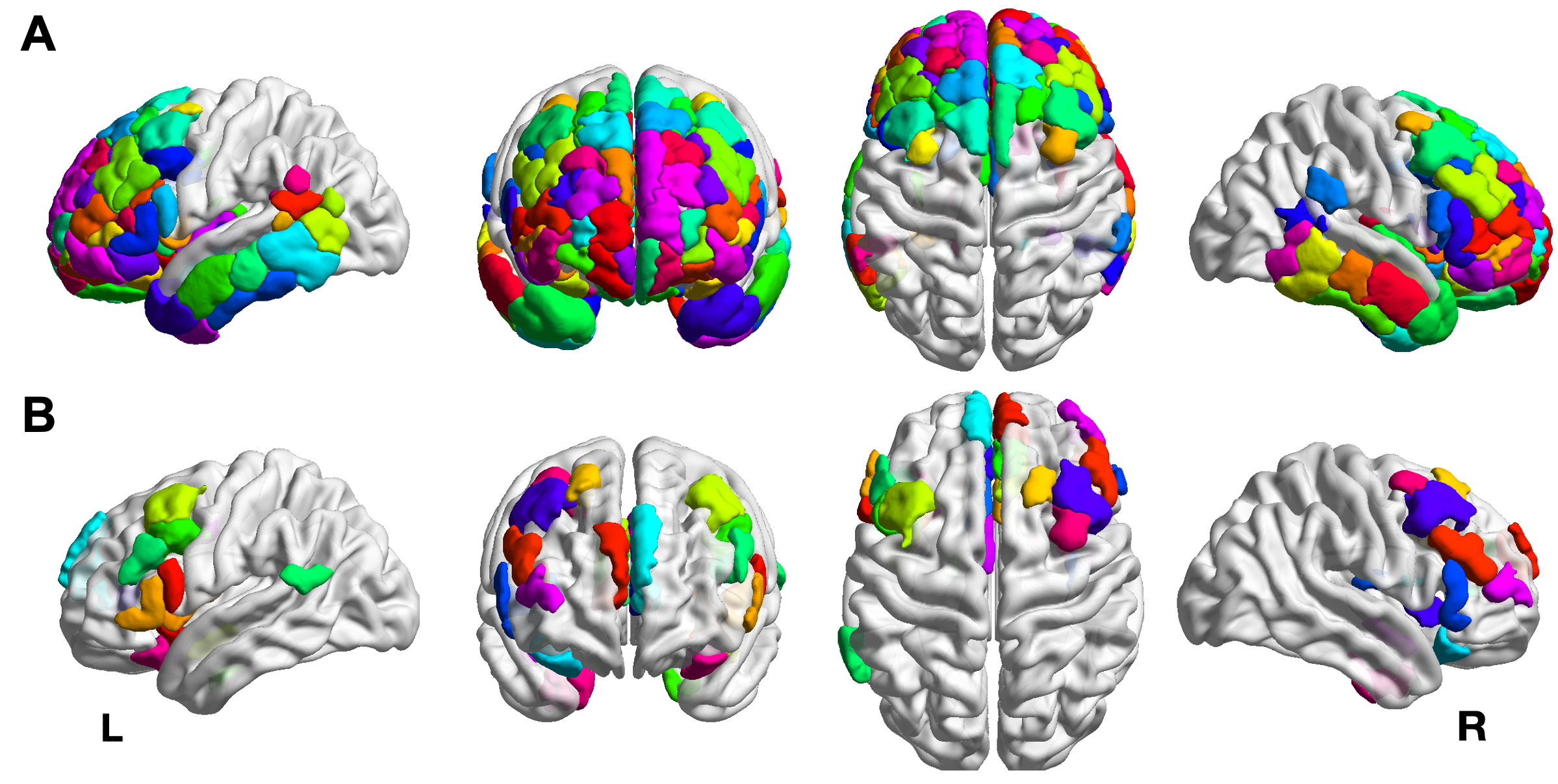


**Supplementary Figure 5. Regional linear associations between brain volume and the autocorrelation-based texture feature (linear regression adjusted for age and sex)**. **(A)** Significant positive associations were observed in 125 out of 160 ROIs (*P_FDR_* < 0.05) without adjusting for group. **(B)** After adjusting for group (healthy control (n =33) and behavioral variant frontotemporal dementia (n = 33)) as a covariate, the number of significant ROIs decreased to 33 (*P_FDR_* < 0.05). Each color represents brain regions according to the Glasser atlas.


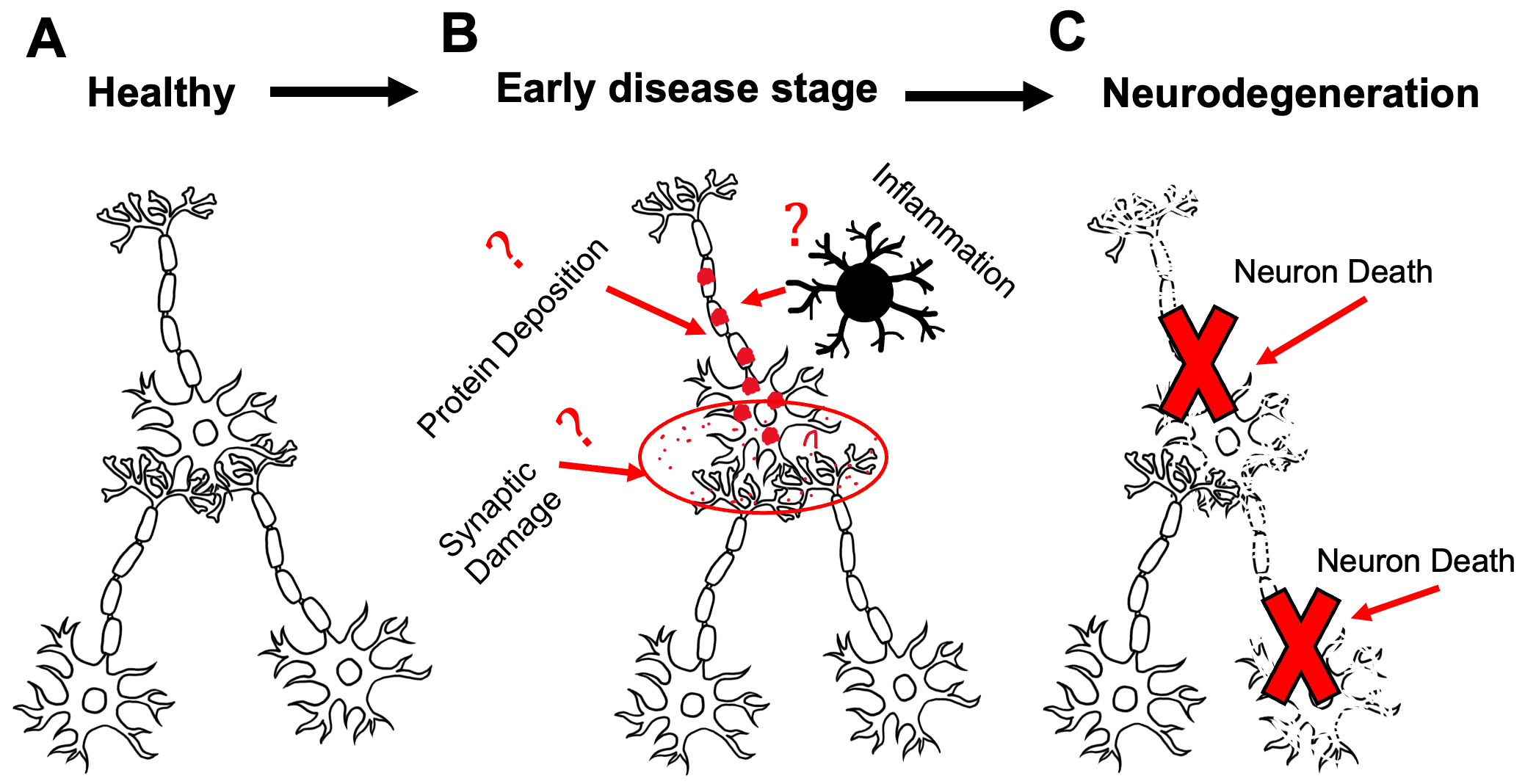


**Supplementary Figure 6. Illustration of neuronal changes across disease stages.** In healthy controls, neurons are structurally intact with normal synaptic connections. In the early stage of neurodegeneration, subtle microstructural alterations may occur due to abnormal protein deposition (e.g., tau, TDP-43), synaptic dysfunction, or inflammation. In advanced neurodegeneration, progressive neuronal loss and structural degradation become more pronounced. Texture analysis may capture these early microstructural changes before significant volumetric atrophy is detectable.
